# Ozzy Osbourne and Parkinson's disease: from darkness to awareness

**DOI:** 10.1055/s-0046-1823655

**Published:** 2026-06-25

**Authors:** Luis F. Fabrini Paleare, José Luiz Pedroso, Flávia de Paiva Santos Rolim, Natália R. Karpejany, Carlos Henrique Ferreira Camargo, Hélio A. G. Teive, Gustavo L. Franklin

**Affiliations:** 1 Pontifícia Universidade Católica do Paraná, Escola de Medicina, Departamento de Medicina Interna, Curitiba PR, Brazil.; 2 Universidade Federal de São Paulo, Departamento de Medicina Interna, São Paulo SP, Brazil.; 3 Universidade de Fortaleza, Escola de Medicina, Fortaleza CE, Brazil.; 4 Hospital Geral de Fortaleza, Departamento de Neurologia, Fortaleza CE, Brazil.; 5 Universidade Federal do Piauí, Hospital Universitário, Teresina PI, Brazil.; 6 Universidade Federal do Paraná, Programa de Pós-Graduação em Medicina Interna, Disciplina de Doenças Neurodegenerativas, Curitiba PR, Brazil.; 7 Universidade Federal do Paraná, Departamento de Clínica Médica, Curitiba PR, Brazil.

**Keywords:** Parkinson Disease, Parkinsonian Disorders, Genetics, Stem Cell Transplantation, Music

## Abstract

Ozzy Osbourne, the legendary frontman of Black Sabbath, publicly revealed his diagnosis of Parkinson's disease (PD) in 2020, offering visibility to a complex neurodegenerative condition. His case, later linked to a mutation in the *PARK2* (parkin) gene, presented atypically with a later age of onset, contributing to ongoing discussions about the phenotypic variability of genetic forms of PD. Beyond medical narratives, Osbourne's openness and philanthropy—culminating in a benefit concert that raised $190 million for Parkinson's and pediatric charities—played a transformative role in destigmatizing the disease. This article explores the scientific and social impact of Osbourne's disclosure, highlighting the role of *PARK2* in mitochondrial homeostasis, synaptic integrity, and tumor suppression. We also examine his pursuit of experimental stem cell therapy, discussing its scientific basis, ethical considerations, and current clinical research landscape.

## INTRODUCTION

Parkinson's disease (PD) is the second most common neurodegenerative disorder. Its prevalence has risen, positioning PD among the leading causes of neurological disability globally. 1 Despite the advances in pathogenesis and treatment, social stigma persists. Public disclosures by prominent figures have helped demystify PD and raise awareness. When rock musicians Neil Diamond (2018), Ozzy Osbourne (2020), and Morten Harket (2025) revealed their diagnoses, their statements helped normalize PD. 2 3 4 Such disclosures may lessen the sense of isolation experienced by individuals living with PD and facilitate greater acceptance of the condition in both clinical and social contexts. 4

In this historical perspective, we examine the relationship between Ozzy Osbourne and PD, focusing on how his public journey contributed to broader awareness of the disease and its personal, clinical, and societal dimensions.

## OZZY OSBOURNE – A SHORT BIOGRAPHY

John Michael Osbourne was born on December 3, 1948, in Birmingham, England. Known for his eccentric style and troubled youth, he attempted suicide several times, dropped out of school at 15, and faced early challenges, including a brief stint in prison for theft at age 17, before embarking on a varied path that would eventually lead to musical fame. 2

**Figure 1:**
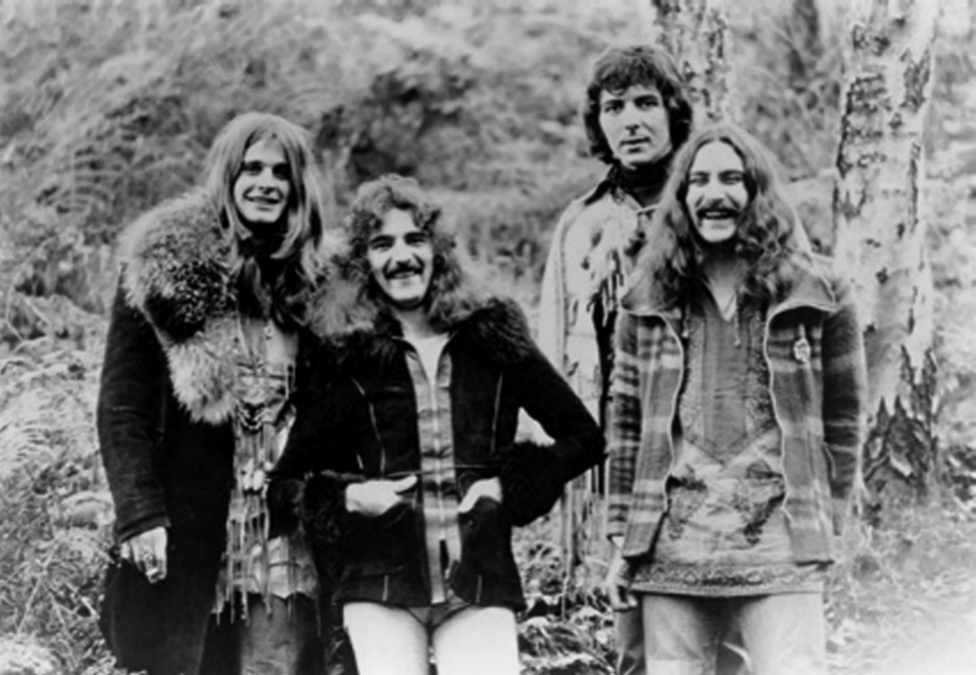
Source: Wikimedia Commons. Ozzy Osbourne, and the original lineup of Black Sabbath, in 1973.

In 1968, Osbourne joined the band that would eventually become Black Sabbath ( Figure 1 ). Their 1970 debut, “Black Sabbath”, marked the start of heavy metal. After leaving the band in 1979, Ozzy pursued a successful solo career with 13 studio albums, beginning with “Blizzard of Ozz” in 1980. 2

In the early 2000s, Ozzy starred in “The Osbournes,” a reality show featuring his family. He was inducted into the Rock and Roll Hall of Fame with Black Sabbath (2006) and as a solo artist (2024). 2

**Figure 2:**
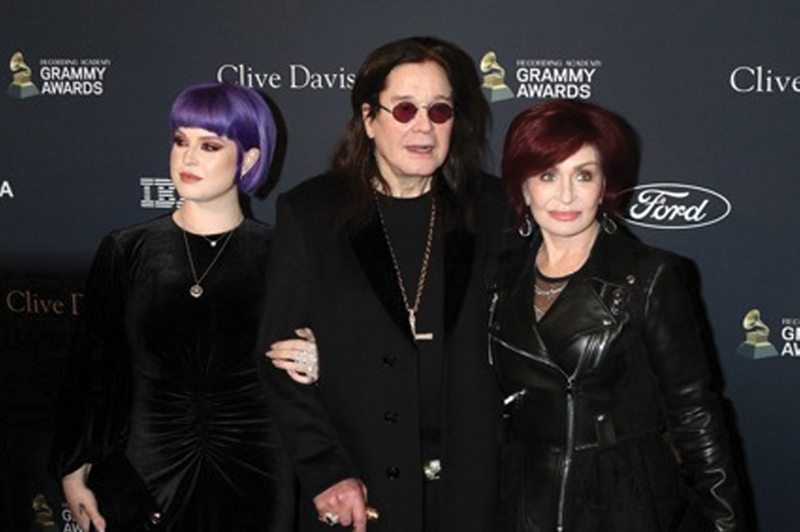
Source: Stock photos by Vecteezy.com. Ozzy Osbourne attending the 2020 Clive Davis Pre-Grammy Party at the Beverly Hilton Hotel on January 25, 2020, in Beverly Hills, CA, the year he publicly disclosed his diagnosis.

In January 2020, he publicly revealed he had been diagnosed with PD, discussing his medical condition and its impact on his daily life ( Figure 2 ). 2

## OZZY AND PARKINSON'S DISEASE

In his autobiographical book “I Am Ozzy”, 2 published in 2009, Ozzy Osbourne reports the onset of tremor, speech impairment, stuttering, and fatigue in 2003, which led to multiple medical evaluations. During this period, he was taking up to 42 medications daily, including sedatives, antidepressants, amphetamines, antiseizure drugs, and antipsychotics, without clinical improvement. He subsequently underwent neurological assessment with Dr. Allan Ropper in Boston. Owing to an atypical clinical presentation and the potential confounding effects of long-standing substance abuse, the diagnosis of PD was initially considered uncertain, and his condition was broadly classified as a parkinsonian syndrome. 2 Several years later, Osbourne and his wife, Sharon Osbourne, publicly disclosed during an interview on *Good Morning America* that a mutation in the *PARK2* gene had been identified. 5 Importantly, this genetic diagnosis has never been reported in a peer-reviewed scientific publication and remains known solely through media statements. This raises questions about the accuracy of the reported onset of symptoms and the possibility of a more complex, late-onset phenotype in his case.

## THE EXPERIMENTAL USE OF STEM CELLS IN PARKINSON'S DISEASE

After diagnosis, Osbourne faced health issues including falls, spinal surgeries, and motor impairments, some as disease complications. 2 In 2020, he underwent experimental stem cell therapy in Panama, followed by a second infusion 3 months later. Initially, Ozzy and his family reported improved motor function, speech, and muscle strength after the first cycle. However, symptoms progressed, and he did not repeat the procedure. 2 3 4

Stem cell therapy for PD is still considered experimental. It is not approved by major regulatory bodies such as standard clinical guidelines, the Food and Drug Administration (FDA), or United Kingdom authorities. 4 This is because its long-term safety and effectiveness have not been established. Current medical studies show that stem cell therapy for PD is moving from preclinical research into early clinical trials, with the main goal of replacing lost dopaminergic neurons in the midbrain. 6 7 8 9 10

Phase I/IIa clinical trials are in progress, using both embryonic and induced pluripotent stem cells. These cells are turned into dopaminergic neuron precursors and transplanted to replace the neurons lost in the substantia nigra. 6 The main results so far relate to the safety and feasibility of these procedures. There is also early evidence that these transplanted cells can survive and integrate, first in animal models and, more recently, in humans. 6 7 8 9 10

However, clear long-term improvement in symptoms has not yet been proven. Ongoing research is still investigating key challenges, including graft-induced dyskinesias, immune rejection (which may require immunosuppression), limited durability of transplanted cells, uncertain outcomes for non-motor symptoms, and ethical issues related to the use of embryonic stem cells. These treatments for PD are still in the research stage and not part of routine care. 6 7 8 9 10

## PARKINSON'S DISEASE AND THE *PARK2* GENE

As a complex neurodegenerative disorder, PD is typically attributed to the interaction between genetic susceptibility and environmental factors, rather than a single causative mutation. 11 However, monogenic forms of PD have been identified, arising from pathogenic variants with either an autosomal dominant inheritance pattern—such as those in *LRRK2* , *SNCA* , and *VPS35* —or an autosomal recessive inheritance pattern, including variants in *DJ-1* , *PINK1* , and *PARK2* . 11

The *PARK2* gene, also known as *Parkin* , is located on the long arm of chromosome 6 (6q25.2–q27). It was first described in 1998 as responsible for an autosomal recessive form of juvenile parkinsonism, with onset before the age of 50. 12 13 14 Its discovery followed decades of clinical and genetic investigation, particularly in Japanese families with early-onset parkinsonism and recessive inheritance patterns. 13 14

## BACK TO THE BEGINNING

Ozzy Osbourne's final Black Sabbath performance, the “Back to the Beginning” concert on July 5, 2025, at Villa Park in Birmingham, showed his strong ties to his birthplace and philanthropy. Ozzy declared that all proceeds from the event would be donated to charity. The concert raised approximately $190 million, to be shared equally among Cure Parkinson's, Birmingham Children's Hospital, and Acorn Children's Hospice. Beyond this monumental donation, Ozzy and his family's public engagement around PD—through candid interviews and stories of his battle with the illness—helped dismantle stigma and build solidarity among patients, caregivers, and researchers.

Ozzy Osbourne died July 22, 2025, at age 76. His life included prolonged instability and substance abuse, complicating his clinical history. In later years, publicly acknowledging his PD increased disorder visibility and encouraged open discussion on its complexity and patient experience.
